# Effect of goal-directed fluid therapy on renal function in critically ill patients: a systematic review and meta-analysis

**DOI:** 10.1080/0886022X.2022.2072338

**Published:** 2022-05-10

**Authors:** Cong-Cong Zhao, Yan Ye, Zhi-Qiang Li, Xin-Hui Wu, Chai Zhao, Zhen-Jie Hu

**Affiliations:** a Department of Intensive Care Unit, The Fourth Hospital of Hebei Medical University, Shijiazhuang, China; b Department of Intensive Care Unit, North China University of Science and Technology Affiliated Hospital, Tangshan, China

**Keywords:** Acute kidney injury, fluid therapy, critical care, systematic review, meta-analysis

## Abstract

**Objective:**

To evaluate whether goal-directed fluid therapy (GDFT) reduces the risk of renal injury in critical illness.

**Methods:**

MEDLINE *via* PubMed, EMBASE, CENTRAL and CBM was searched from inception to 13 March 2022, for studies comparing the effect of GDFT with usual care on renal function in critically ill patients. GDFT was defined as a protocolized intervention based on hemodynamic and/or oxygen delivery parameters. A fixed or random effects model was applied to calculate the pooled odds ratio (OR) based on heterogeneity through the included studies.

**Results:**

A total of 28 studies with 9,019 patients were included. The pooled data showed that compared with usual care, GDFT reduced the incidence of acute kidney injury (AKI) in critical illness (OR 0.62, 95% confidence interval (CI) 0.47 to 0.80, *p*< 0.001). Sensitivity analysis with only low risk of bias studies showed the same result. Subgroup analyses found that GDFT was associated with a lower AKI incidence in both postoperative and medical patients. The reduction was significant in GDFT aimed at dynamic indicators. However, no significant difference was found between groups in RRT support (OR 0.88, 95% CI 0.74 to 1.05, *p*= 0.17). GDFT tended to increase fluid administration within the first 6 h, decrease fluid administration after 24 h, and was associated with more vasopressor requirements.

**Conclusions:**

This meta-analysis suggests that GDFT aimed at dynamic indicators may be an effective way to prevent AKI in critical illness. This may indicate a benefit from early adequate fluid resuscitation and the combined effect of vasopressors.

## Introduction

Acute kidney injury (AKI) is a clinical syndrome due to an abrupt decrease in kidney function and is typically diagnosed by increased creatinine, decreased urine output, or both [1]. It is a common and serious complication in critically ill patients and is associated with increases in hospitalization cost, morbidity, and mortality [2,3]. It should be noted that just with the occurrence of AKI, short- and long-term survival will be significantly reduced for patients with AKI regardless of its severity and evolution [4]. Therefore, the prevention of AKI is crucial in critically ill patients.

AKI prevention is a multimodal clinical algorithm based on protocolized volume status and perfusion pressure optimization [5], which require adequate renal blood flow. Unfortunately, routine hemodynamic measurements, such as the mean blood pressure (MAP) and central venous pressure (CVP), are poor predictors of volume status and renal blood flow in critical illness. The end points of fluid resuscitation are uncertain and challenging, which leads to the development of protocolized hemodynamic resuscitation. This goal-directed fluid therapy (GDFT) approach uses intensive monitoring, including some measures of hemodynamics (such as MAP, CVP, cardiac output (CO), and stroke volume (SV)) and oxygen delivery parameters (such as oxygen delivery, central venous oxygenation (ScvO_2_) or mixed venous oxygenation).

GDFT reduced the risk of perioperative complications [6,7], including renal injury [8]. A recent meta-analysis found that GDFT improved renal perfusion and oxygenation in high-risk patients undergoing major abdominal and orthopedic surgery [9]. In addition, previous studies have suggested that GDFT is associated with a decrease in AKI incidence in critical illness [10–12]. However, some studies found no beneficial effect of GDFT on renal function [13–15]. Furthermore, different kinds of patients, protocolized goals, and study designs make it difficult to provide specific recommendations for GDFT.

Hence, this meta-analysis aimed to evaluate the effects of GDFT on renal function in critically ill patients. In particular, we tried to clarify which protocolized goals are effective, what kinds of patients can benefit from them, and the roles of fluids and vasopressors in this approach.

## Methods

### Protocol and registration

This systematic review was performed according to the Preferred Reporting Items for Systematic Reviews and Meta-Analyses (PRISMA) guidelines [16]. The protocol of this work was registered in the PROSPERO database (CRD42021233518).

### Search strategy

We searched the MEDLINE *via* PubMed, EMBASE, CENTRAL and CBM databases (from inception to 13 March 2022) using the terms: (“intensive care” or “emergency” or “critical illness”) AND (“fluid resuscitation” or “fluid therapy”) AND (“goal-directed” or “goal-oriented” or “target-directed”). There were no language limits on eligibility. The Supplementary Material 1 shows the search strategy in more detail.

### Study selection

Two investigators (CCZ, YY) independently determined whether eligible studies met the following PICOS criteria: 1) Population: adult patients (age ≥18 years) treated at an intensive care unit or emergency department; 2) Intervention: protocolized and based on hemodynamic and oxygen delivery parameters; 3) Control: usual care, defined as conventional treatments that were at the discretion of the clinicians. Monitoring by CVP or MAP measurements was allowed. 4) Outcomes: The primary outcome was the incidence of AKI at any time point during hospitalization, whichever definition of AKI was adopted. Secondary outcomes were renal replacement therapy (RRT) support, fluid administration, and vasopressor requirements. 5) Type of studies: randomized controlled trials (RCTs), quasi-RCTs, and prospective and retrospective controlled trials.

The exclusion criteria were as follows: 1) lack of a protocolized intervention; 2) lack of a baseline condition or control group; 3) lack of data on any renal outcome: AKI and RRT; and 4) no original studies, case reports, case series, animal studies, *in vitro* studies, and studies without full text.

### Data extraction

The initial and full-text reviews and data extraction from the included studies were performed independently by two authors (CCZ, YY). The kappa coefficient was calculated as a measure of agreement about study selection and quality appraisal. Any discrepancies were resolved by the third author (ZQL), and a decision was reached by consensus.

Data were collected using a predesigned form. For each study, the following information was extracted: publication (last name of the first author, year of publication), participant characteristics (including patient source, diagnosis, demographic data, clinical setting, and number of patients), targets used in the GDFT protocol (including MAP, CVP, CO/CI, SV, stroke volume variation (SVV), pulse pressure variation (PPV), oxygen delivery parameters *et al.*), study design, and outcome data.

### Assessment of risk of bias

Two authors (CCZ, YY) independently assessed the risk of bias to evaluate the quality of the included studies. The Cochrane Collaboration tool [17] was used for RCTs, and the ROBINS-I tool (Risk of Bias in Nonrandomized Studies of Interventions) [18] was used for non-RCTs. Funnel plot was used to evaluate publication bias.

### Statistical analysis

SPSS 25.0 was used to calculate the kappa coefficient. Data analysis was conducted using RevMan 5.3. The results are presented as forest plots using odds ratios (ORs) for dichotomous data and the mean difference (MD) for continuous data. All estimates were provided with 95% confidence intervals (CIs). Heterogeneity was assessed by Cochran’s Q statistic and the I^2^ test. A *P* value >0.1 or I^2^ statistic below 50% indicated low levels of heterogeneity. In these cases, a fixed-effect model was used. Otherwise, a random-effects model (Mantel–Haenszel method) was selected. Sensitivity analysis with only low risk of bias studies was considered if the pooled data of the primary outcome had significant heterogeneity. Several subgroup analyses were performed for the primary outcome according to population (postoperative and medical patients), GDFT protocol (early goal-directed therapy (EGDT) [11], dynamic indicators (defined as the variation of certain indicators in the GDFT protocol), and other protocols), and the design of the trial (RCT and non-RCT). *p*< 0.05 indicated statistical significance.

## Results

**Figure 1.**
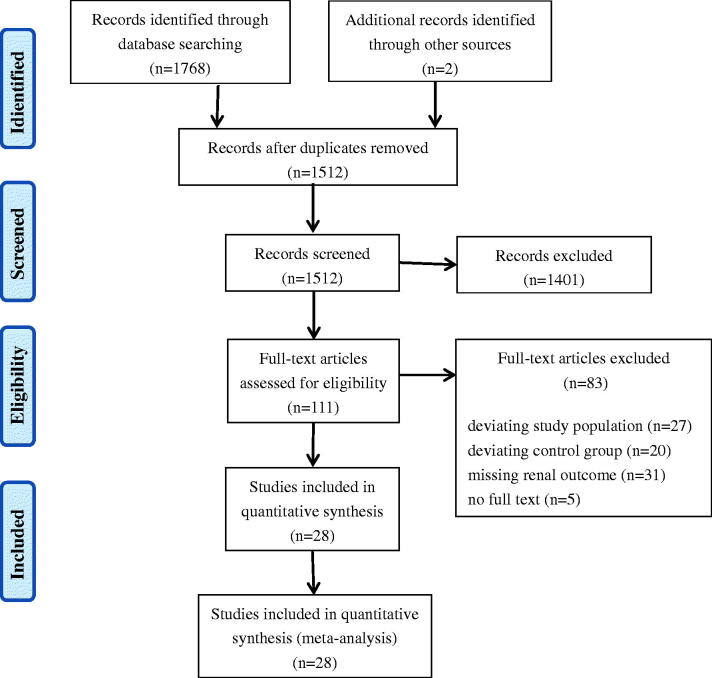
Study selection flow diagram according to the PRISMA guidelines.

The search strategy identified 1,768 unique publications, and 2 additional records were identified from reference lists. After excluding duplicates (*n* = 258) and screening titles and abstracts (*n* = 1,512), 111 studies were assessed in full text for eligibility (Figure 1). Following full-text review, a total of 28 studies met the inclusion criteria (kappa = 0.858, *p*< 0.01). Among these, three studies were reported in Chinese [19–21], and all the other studies were reported in English [10,14,22–44].

**Figure 2.**
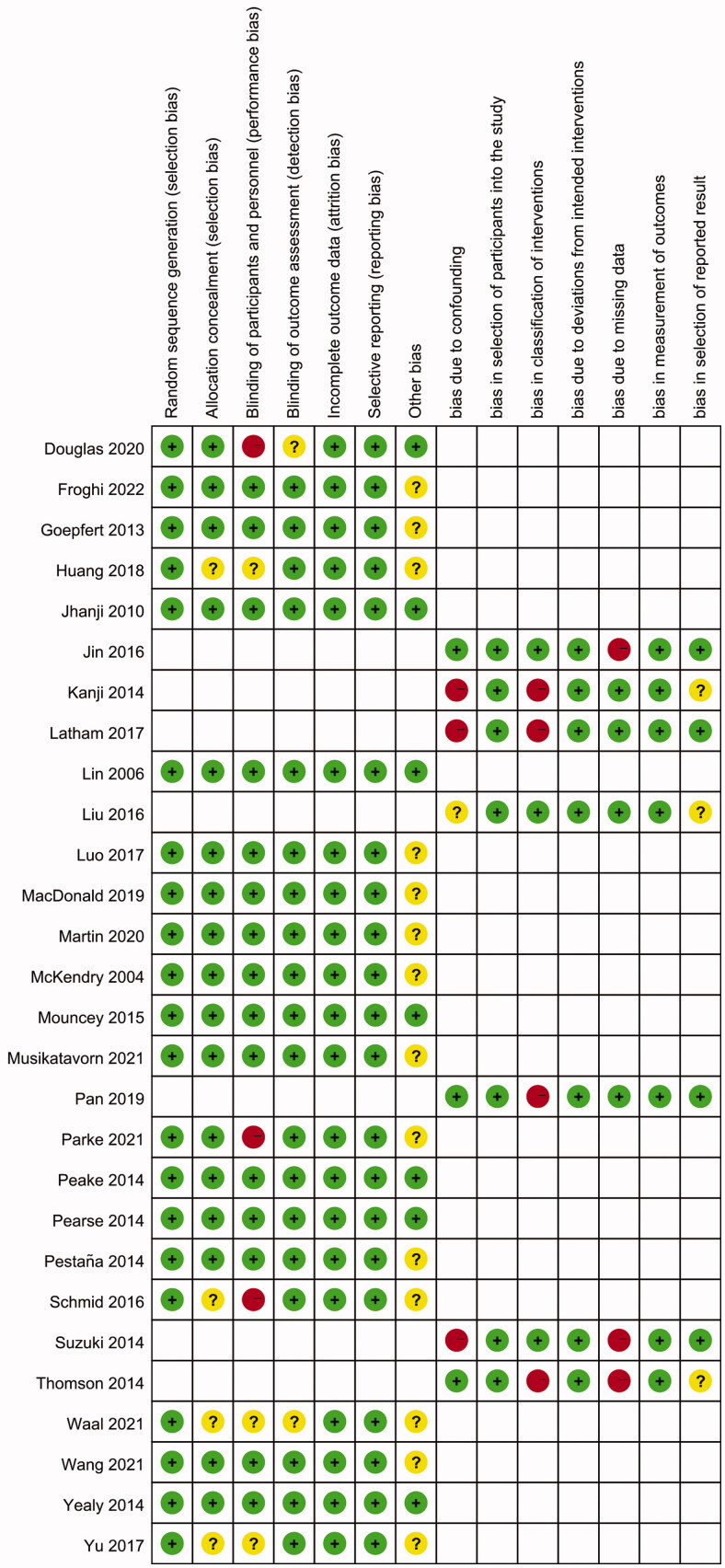
Risk of bias summary assessments for included studies.

The characteristics of the included studies are summarized in Table 1. In total, the included studies comprised 9,019 patients, and the number of patients per study was 48 to 1,591. Of the 28 included studies, 21 studies were RCTs, and the other seven were not. The risk of bias assessments for RCTs and non-RCTs are shown in Figure 2 (kappa = 0.766, *p*< 0.01). The green, red and yellow colors indicate a low risk of bias, a high risk of bias, and an unclear risk of bias, respectively. Studies with more than or equal to five green plus were considered low risk of bias studies. Among the 28 included studies, all except for five studies [27,28,38,40,42] had a low risk of bias overall.

**Table 1.** Characteristics of studies included in this meta-analysis.

Studies	Country	Design	Department	Population	No. of patients (GDFT vs. Control)	GDFT proposal	Control	Outcomes
McKendry et al.2004 [32]	United Kingdom	RCT	Surgical ICU	post-surgery	174 （89 vs. 85）	SVI >35 ml/m^2^ & increase ≤ 10% after fluid challenge	usual care	AKI, fluids administration
Lin et al. 2006 [10]	China	RCT	Medical ICU	septic shock	224 (108 vs. 116)	EGDT without ScvO_2_	usual care	AKI, fluids administration, vasopressor requirement
Jhanji et al. 2010 [26]	United Kingdom	RCT	ICU	post-surgery	90 (45 vs. 45)	SV increase <10% after fluid challenge	CVP rise ≥2 mmHg	AKI, fluids administration, vasopressor requirement
Goepfert et al.2013 [24]	Germany	RCT	ICU	post-surgery	92(46 vs. 46)	SVV ≤ 10%	CVP > 8 or MAP > 65 mmHg	AKI, fluids administration
Kanji et al. 2014 [27]	Canada	prospective before-after study	ICU	undifferentiated shock	220(110 vs. 110)	IVC collapsibility < 15%	CVP 8–12 mmHg	AKI, vasopressor requirement
Peake et al. 2014 [25]	Australia & New Zealand	RCT	Emergency department	early septic shock	1591(793 vs. 798)	EGDT	usual care	RRT, fluids administration, vasopressor requirement
Pearse et al. 2014 [36]	United Kingdom	RCT	ICU	post-surgery	734 (368 vs 366)	CO-guided hemodynamic therapy algorithm	usual care	AKI, fluids administration, vasopressor requirement
Pestaña et al. 2014 [37]	Spain	RCT	ICU	post-surgery	142 (72 vs. 70)	MAP ≥65 mmHg & CI ≥2.5 L/min/m^2^	usual care	AKI, fluids administration, vasopressor requirement
Suzuki et al. 2014 [39]	Australia	prospective before-after study	ICU	post-surgery	98 (53 vs. 45)	PPV < 13%	usual care	AKI, fluids administration
Thomson et al. 2014 [40]	United Kingdom	prospective cohort study	Cardiothorac-ic ICU	post-surgery	264 (123 vs. 141)	SV increase <10% after fluid challenge	standard therapy	AKI, RRT, fluids administration
Yealy et al. 2014 [14]	United States	RCT	Emergency department	septic shock	895 (439 vs. 456)	EGDT	usual care	AKI, RRT, fluids administration, vasopressor requirement
Mouncey et al. 2015 [33]	United Kingdom	RCT	Emergency department	early septic shock	1251 (625 vs. 626)	EGDT	usual care	RRT, fluids administration, vasopressor requirement
Liu et al. 2016 [19]	China	prospective before-after study	ICU	Moderate brain injury& traumatic shock	98 (48 vs. 50)	EGDT	usual care	AKI, fluids administration
Jin et al. 2016 [22]	China	retrospective before-after study	ICU	post-surgery	232 (131 vs. 101)	CVP 10–12 mmHg; CI ≥ 2.4 L/min/m^2^; lactate <4 mmol/L; SvO_2_ >65%	MAP 65-100 mmHg	AKI, fluids administration
Schmid et al. 2016 [38]	Germany	RCT	ICU	post-surgery	180 (92 vs. 88)	MAP >70 mmHg, CI >2.5 L/min/m^2^; GEDI >800 mL/m^2^; ELWI <10 ml/kg	usual care	AKI, RRT, fluids administration
Latham et al. 2017 [28]	United States	retrospective cohort study	Medical or Transplant ICUs	post-surgery	191 (100 vs. 91)	SVI increased <10% after fluid challenge	usual care	RRT
Luo et al. 2017 [29]	China	RCT	ICU	severe sepsis &septic shock	145 (73 vs. 72)	CI >2.5 L/min/m^2^, SVV <15%	usual care	AKI, RRT, fluids administration, vasopressor requirement
Yu et al. 2017 [41]	China	RCT	ICU	COPD with septic shock	71 (34 vs. 37)	GEDI ≥800mL/m^2^	usual care	RRT, fluids administration
Huang et al. 2018 [20]	China	RCT	ICU	hypovolemic shock	48 (25 vs. 23)	EGDT	CVP 8–12 mmHg	AKI, fluids administration, vasopressor requirement
MacDonad et al. 2019 [30]	United Kingdom	RCT	ICU	post-surgery	287 (144 vs. 143)	CO-guided hemodynamic therapy algorithm	usual care	AKI
Pan et al. 2019 [21]	China	prospective cohort study	ICU	post-surgery	171 (103 vs. 68)	CI >2.5 L/min/m^2^;GEDVI >700 mL/m^2^ or ITBVI > 850 ml/m^2^; EVLWI＜10 mL/kg; MAP > 65 mmHg	CVP8-10, MAP > 65 mmHg	AKI, RRT
Douglas et al. 2020 [23]	United States & United Kingdom	RCT	Emergency department & ICU	sepsis or septic shock	150 (102 vs 48)	SV increase <10% after passive leg raise	usual care	RRT
Martin et al. 2020 [31]	United Kingdom	RCT	ICU	post-surgery	60 (30 vs. 30)	SVV ≤10%	usual care	RRT, fluids administration
Musikatavorn et al. 2021 [34]	Thailand	RCT	Emergency department	septic shock	220 (101 vs. 101)	IVC collapsibility <40% (non-MV) or IVC distensibility <18% (MV)	usual care	AKI, RRT, fluids administration, vasopressor requirement
Parke et al. 2021 [35]	Australian & New Zealand	RCT	Surgical ICU	post-surgery	715 (358 vs. 357)	CI >2.5 L/min/m^2^, SVV <13%	usual care	AKI, RRT, fluids administration, vasopressor requirement
Waal et al. 2021 [42]	China	RCT	Surgical ICU	post-surgery	482 (234 vs. 248)	age-dependent target for CI & SVV and PLR	usual care	AKI
Wang et al. 2021 [43]	Netherlands	RCT	PACU /ICU	post-surgery	134 (66 vs. 68)	Bioelectrical impedance analysis	usual care	AKI, fluids administration
Froghi et al. 2022 [44]	United Kingdom	RCT	ICU	post-surgery	60 (30 vs. 30)	SV rise ≤10%, SV drop ≤10%	usual care	AKI, fluids administration

AKI: acute kidney injury; CO: cardiac output; CI: cardiac index; COPD: chronic obstructive pulmonary disease; CVP: central venous pressure; EGDT: early goal directed therapy; EVLWI extravascular lung water index; ICU: intensive care unit; IVC: Inferior Vena Cava; GDFT: goal-directed fluid therapy; GEDI Global end-diastolic index; MAP: mean blood pressure; MV mechanical ventilation; No. : number; PACU: Post-Anesthesia Care units; PLR: passive leg raising; PV: pulse pressure variation; RCT: randomized controlled trial; RRT: renal replacement therapy; ScvO_2_: central venous oxygenation; SvO_2_: mixed venous oxygenation; SV: stroke volume; SVI: stroke volume index.

### Incidence of AKI

**Figure 3.**
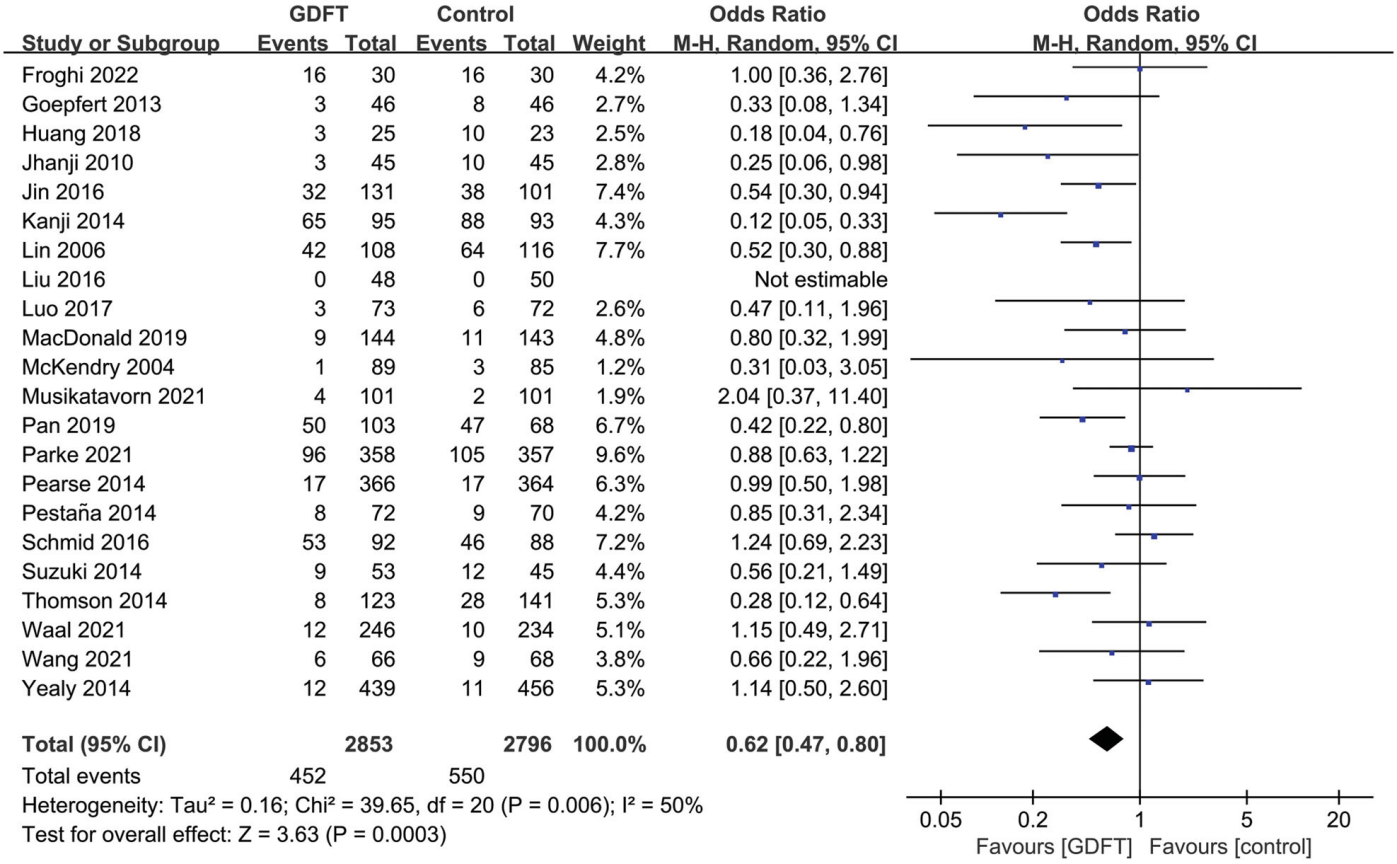
Forest plot of the effect of GDFT on AKI incidence without time limit. AKI: Acute kidney injury; GDFT: goal-directed fluid therapy; M-H: Mantel–Haenszel; CI: confidence interval.

Twenty-two (*n* = 5,649, 2,853 in the GDFT group and 2,796 in the control group) of the 28 included studies reported that the incidence of AKI ranged from 0%–95% with different follow-up times. All but six of the included studies reported a clear definition of AKI. The incidence of AKI was lower in postoperative patients and higher in medical patients. The detailed parameters of AKI, including the morbidity, definition, and follow-up time in each study, are shown in Supplementary Table 1. The pooled data showed that GDFT significantly reduced AKI incidence over usual care (OR 0.62, 95% CI 0.47 to 0.80, *p*< 0.001; Figure 3). The heterogeneity was moderate (I^2^=50%). No sign of significant publication bias was observed (Supplementary Figure 1). The sensitivity analysis considering only low risk of bias studies showed the same result: compared with usual care, GDFT reduced the risk of renal injury in critically ill patients (OR 0.66, 95% CI 0.54 to 0.82, *p*< 0.001; I^2^=13%).

**Figure 4.**
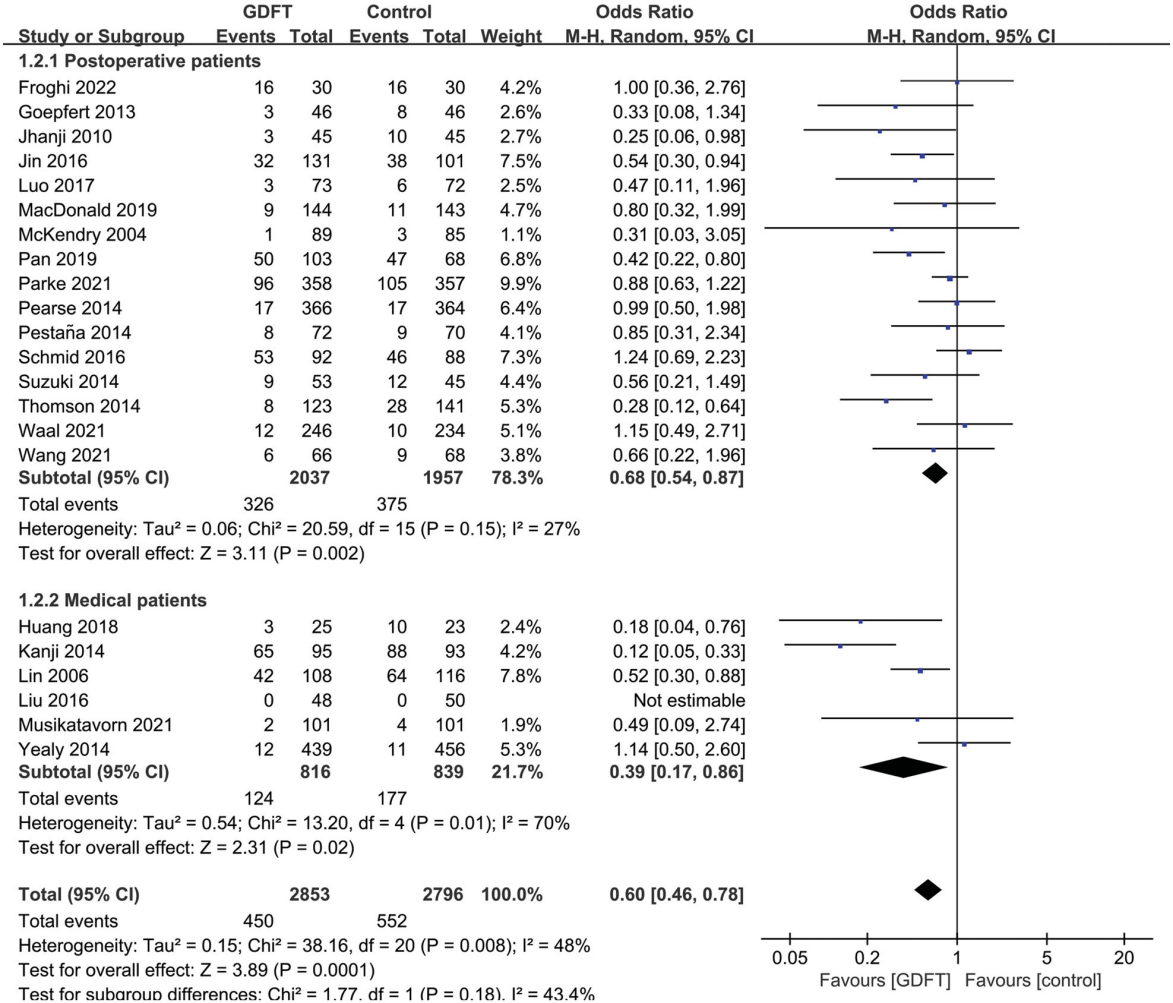
Pooled AKI incidence of subgroup analysis concerning postoperative and medical patients. AKI: Acute kidney injury; M-H: Mantel–Haenszel; CI: confidence interval.

**Figure 5.**
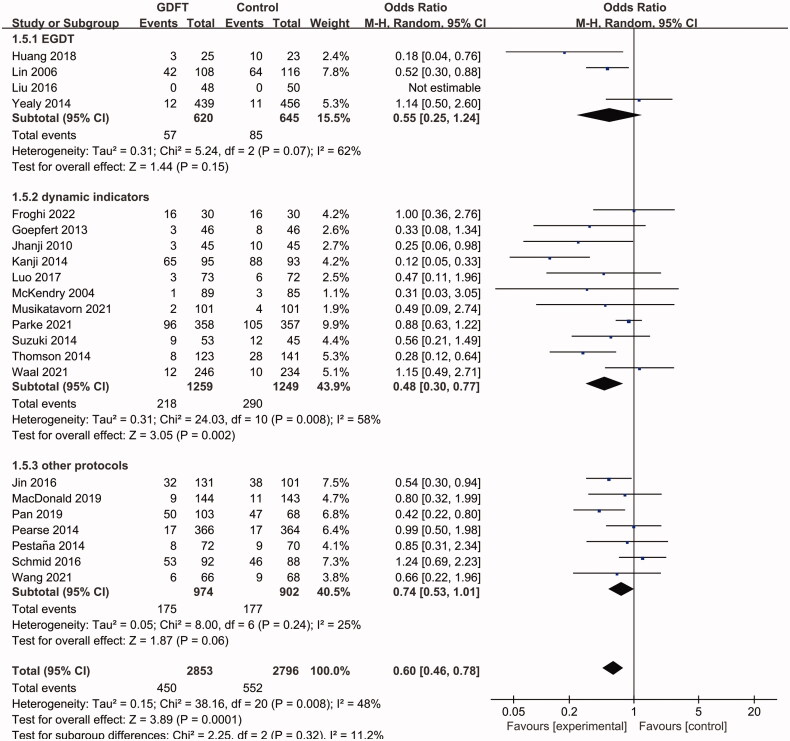
Pooled AKI incidence of subgroup analysis concerning different GDFT protocols, including EGDT, dynamic indicators and other protocols. AKI: Acute kidney injury; GDFT: goal-directed fluid therapy; EGDT: early goal directed therapy; M-H: Mantel–Haenszel; CI: confidence interval.

**Figure 6.**
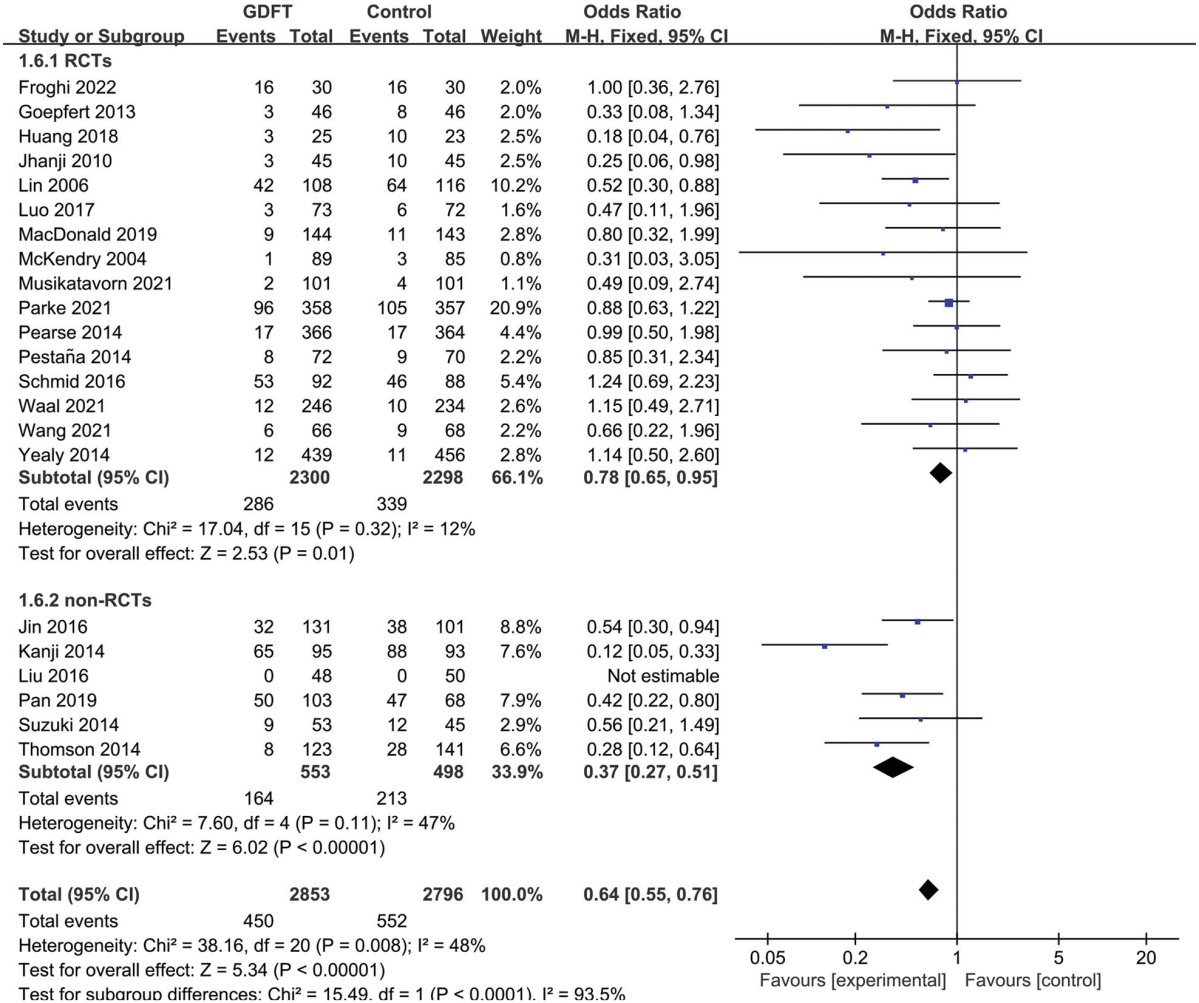
Pooled AKI incidence of subgroup analysis concerning RCTs and non- RCTs. AKI, Acute kidney injury; RCT, randomized controlled trial; M-H, Mantel–Haenszel; CI, confidence interval.

Subgroup analyses showed that in both postoperative and medical patients, the AKI incidences were lower in the GDFT group (postoperative patients: OR 0.68, 95% CI 0.54 to 0.87, *p*= 0.002; medical patients: OR 0.39, 95% CI 0.17 to 0.86, *p*= 0.02; Figure 4). The heterogeneity was low in the subgroup of postoperative patients (I^2^=27%) but higher in the subgroup of medical patients (I^2^=70%). In medical patients, we further performed a subgroup analysis of patients with septic shock, which showed no effect of GDFT on AKI with a lower heterogeneity (OR 0.66, 95% CI 0.39 to 1.14, *p*= 0.14; I^2^=22%). A significant AKI reduction was observed in studies that adopted dynamic indicators (including SVV, PPV, SV change, IVC collapsibility and distensibility) as fluid therapy targets (OR 0.48, 95% CI 0.30 to 0.77, *p*= 0.002; I^2^=58%; Figure 5), instead of these studies with EGDT (OR 0.55, 95% CI 0.25 to 1.24, *p*= 0.15; I^2^=62%; Figure 5) or the other protocols (OR 0.74, 95% CI 0.53 to 1.01, *p*= 0.06; I^2^=25%; Figure 5). In addition, the pooled data from RCTs (OR 0.78, 95% CI 0.65 to 0.95, *p*= 0.01; I^2^=12%; Figure 6) and non-RCTs (OR 0.37, 95% CI 0.27 to 0.51, *p*< 0.001; I^2^=47%; Figure 6) both showed a preventive effect of GDFT on AKI in critically ill patients.

### RRT support

Thirteen (*n* = 5,709, 2,884 in the GDFT group and 2,825 in the control group) of the 28 included studies reported pooled analysis showing GDFT did not decrease the requirement for RRT (OR 0.88, 95% CI 0.74 to 1.05, *p*= 0.17; I^2^=50%; Supplementary Figure 2) in critically ill patients.

### Fluid administration (L)

Of the 28 included studies, 22 (*n* = 11,965, 5,983 in the GDFT group and 5,982 in the control group) reported intravenous fluids at different time points. According to the different record times of fluid administration, the 22 studies were divided into three subgroups: within the initial 6 h, more than 6 h and less than or equal to 24 h, and more than 24 h. The pooled data from 9 studies (*n* = 5,058; 2,526 in the GDFT group and 2,532 in the control group) showed that GDFT tended to increase the volume of fluid administration within the initial 6 h, but there was no significant difference between groups (MD 0.27, 95% CI −0.04 to 0.59, *p*= 0.09; Supplementary Figure 3). The heterogeneity was high. The pooled data from 13 studies (*n* = 2,261; 1,141 in the GDFT group and 1,120 in the control group) showed that GDFT had no effect on fluid administration from 6 to 24 h (MD 0.34, 95% CI −0.14 to 0.81, *p*= 0.16; I^2^=98%; Supplementary Figure 3). However, the cumulative fluid administration in the GDFT group was less than that in the control group after 24 h (*n* = 4,646; 2,316 in the GDFT group and 2,330 in the control group), with high heterogeneity (MD −0.45, 95% CI −0.71 to −0.19, *p*< 0.001; I^2^=90%; Supplementary Figure 3).

### Vasopressor requirements

For this outcome, 12 studies reported the use of vasopressors (*n* = 6,252; 3,116 in the GDFT group and 3,136 in the control group). Compared with the control group, patients in the GDFT group seemed to use more vasopressors, but the difference was not significant (OR 1.23, 95% CI 1.00 to 1.52, *p*= 0.05; I^2^=58%; Supplementary Figure 4).

## Discussion

This systematic review is the first to focus on the effect of GDFT on renal function in critically ill patients. Pooled data demonstrated that the incidence of AKI was reduced by GDFT in critical illness. This result was confirmed by the sensitivity analysis enrolling only low-risk-of-bias trials and the pooled data from RCTs. Subgroup analyses showed that both postoperative and medical patients benefited from GDFT, and the reduction in AKI was significant in GDFT aimed at dynamic indicators. However, GDFT was not associated with a reduction in RRT support.

Critically ill patients are at high risk of AKI, which is closely associated with poor prognosis. The most frequent causes of AKI in critical illness are sepsis, hypovolemia, direct nephrotoxicity, and major surgery [45]. AKI is believed to be initially preventable and reversible [46]. Fluid therapy is a key component of the prevention of AKI, and the aim is to correct hypovolemia and restore organ perfusion in addition to avoiding further nephrotoxic insults. Because routine hemodynamic measurements poorly predict volume status and renal blood flow in critical illness, the GDFT approach has been proposed.

Our study found that in comparison with usual care, GDFT reduced the incidence of AKI in critically ill patients. However, moderate heterogeneity limits the credibility of the results. Several questions remain unanswered, such as kind of patients, protocolized goals, and the impact of the quality of included studies. Therefore, we first performed sensitivity analysis enrolling only low-risk-of-bias trials. This showed the same result with low heterogeneity. Then, subgroup analysis of RCTs further confirmed the main result. In addition, subgroup analyses concerning postoperative versus medical patients and different fluid therapy targets were also performed.

The subgroup analysis of populations showed that GDFT significantly reduced AKI in both postoperative and medical patients. Consistently, a recent meta-analysis, including 65 studies with 9,306 adult patients undergoing major surgery and noncritical illness, which reported a marked decrease in the renal injury rate in the perioperative goal-directed therapy group [9]. It is worth noting that we found the preventive effect on AKI in medical patients. In fact, only six included study populations were medical patients. They were all shock patients, and half of them had septic shock. A further subgroup analysis of patients with septic shock showed no effect of GDFT on AKI. Similarly, a previous multicenter large sample RCT did not find any protective effect of EGDT on renal function in septic shock patients [14]. In patients/animals with septic shock, global renal blood flow is preserved or even increased [47]. In contrast, decreases in glomerular filtration pressure, inflammatory tubules and microvascular injury result in renal dysfunction in sepsis [48]. Therefore, optimizing hemodynamics has limited preventive effects on septic AKI.

The subgroup analysis regarding targets showed that GDFT based on dynamic indicators significantly reduced AKI incidence, rather than EGDT and other protocols. However, the heterogeneity in the ‘dynamic indicators’ subgroup was relatively high and two large studies [27,40] which showed preferable results for GDFT in this subgroup were non-RCTs. These can be a risk of bias and weak the strength of the evidence. About EGDT, it was first introduced by Rivers *et al* and mainly used in patients with sepsis [11]. The effect of EGDT on prognosis, including AKI and mortality, is still controversial [10,14,25,33]. In summary, GDFT aimed at dynamic indicators may be an effective way to protect renal function in critically ill patients.

In addition, there was no effect of GDFT on RRT support. Consistently, a previous meta-analysis found no differences in the RRT rate between the standard EGDT and usual care groups in patients with severe sepsis and septic shock [49]. These results suggested that GDFT did not improve the deterioration of renal function in patients who had already developed AKI. Consistent with this view, one previous study reported that GDFT did not reduce the persistence of AKI beyond 72 h for patients in the early stage of AKI [15]. In summary, the beneficial effect of GDFT on renal function is more significant in patients without AKI.

We further analyzed the role of fluid administration and vasopressor requirements. The results demonstrated that although GDFT tended to increase fluid administration within the initial six hours compared with usual care, it was not substantially different between the groups in the initial 24 h. After 24 h, fluid administration was less common in the GDFT group. In addition, GDFT was associated with more vasopressor requirements. Using vasopressors for a short duration might meet the acute demand for oxygen delivery and limit the volume of fluid administration [7]. Guidelines recommend that accurate and timely fluid therapy improves organ function [50]. Excessive volume expansion is also associated with adverse outcomes, including renal dysfunction [51–53]. GDFT by means of fluids and vasopressors can minimize the time of low perfusion and spare unwarranted fluid therapy, which may contribute to the prevention of AKI.

The strengths of this study include broad search strategy, inclusion of extensive studies and the latest research with high quality of methodology. Moreover, unlike previous meta-analyses focused on the effect of goal-directed therapy on AKI in patients undergoing surgery [9], we are the first to focus on critically ill patients. In addition, we further performed sensitivity analysis and subgroup analyses, generating new hints for practical applications. In fact, all critically ill patients were at risk for AKI. GDFT aimed at dynamic parameters may be more helpful for AKI prevention but does not change the disease course.

This study has a number of limitations. First, the protocols of GDFT varied in the included studies, and the definitions of usual care may be different in different areas. This led to relatively high heterogeneity, although the results remained consistent across sensitivity and subgroup analyses. Second, most of the included studies lacked baseline kidney function, that is, chronic kidney disease. Third, the included studies varied in definitions, and timeframes of AKI incidence were another limitation. Last, there may be potential publication bias.

## Conclusions

In conclusion, this meta-analysis suggests that GDFT reduced the incidence of AKI in critical illness, including postoperative and medical patients. Sensitivity analysis enrolling only trials with a low risk of bias and subgroup analysis of RCTs confirmed this result. The reduction was significant in GDFT based on dynamic indicators, rather than EGDT and other protocols. However, there was no difference in RRT support between the groups. Fluid administration seemed to be higher in the GDFT group within the first 6 h but lower after 24 h. Moreover, GDFT was associated with more vasopressor requirements. Prompt, targeted resuscitation combined with fluid and vasopressors may contribute to the prevention of AKI.

## Supplementary Material

Supplemental Material

Supplemental Material

Supplemental Material

Supplemental Material

Supplemental Material

Supplemental Material

## Data Availability

The authors confirm that the data supporting the findings of this study are available within the article.
