# Supplementary figures and images for: Effect of goal-directed fluid therapy on renal function in critically ill patients: a systematic review and meta-analysis
Source: Ren Fail. 2022 May 10;44(1):777–89. doi: 10.1080/0886022X.2022.2072338 (PMC9103701; doi:10.1080/0886022X.2022.2072338)

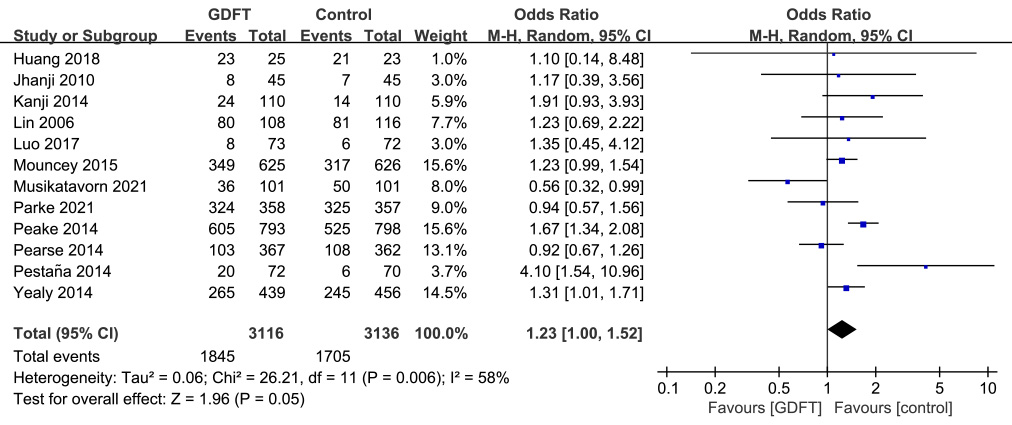

Supplement: Supplemental Material [file IRNF_A_2072338_SM1570.jpg]

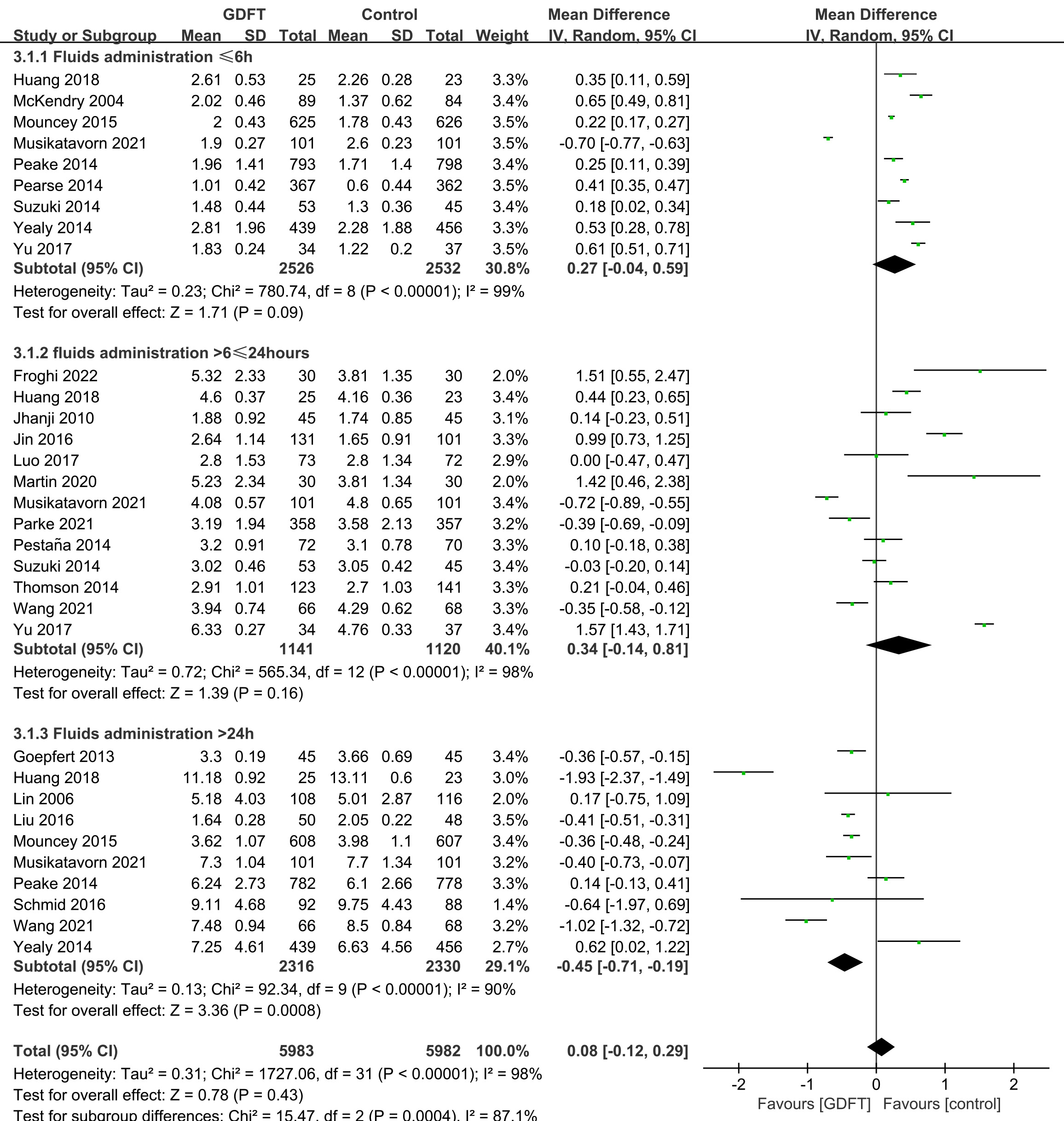

Supplement: Supplemental Material [file IRNF_A_2072338_SM1566.jpg]

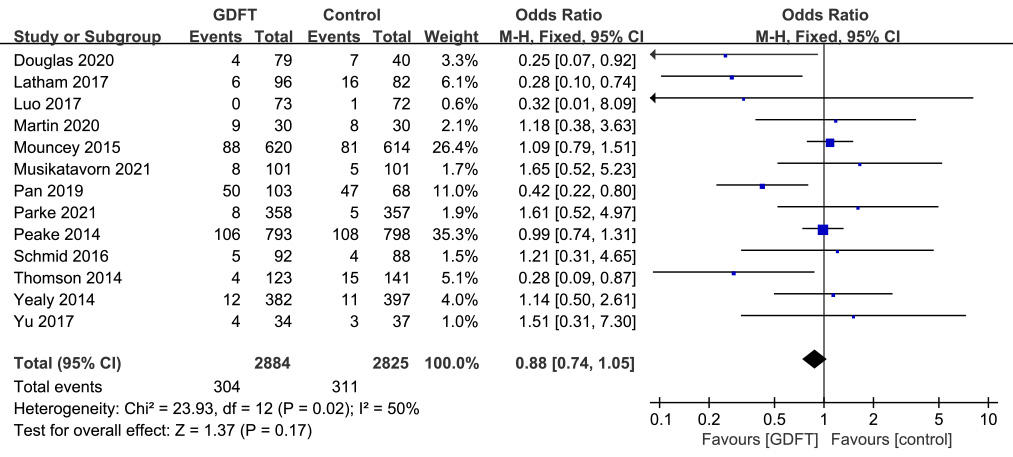

Supplement: Supplemental Material [file IRNF_A_2072338_SM1561.jpg]

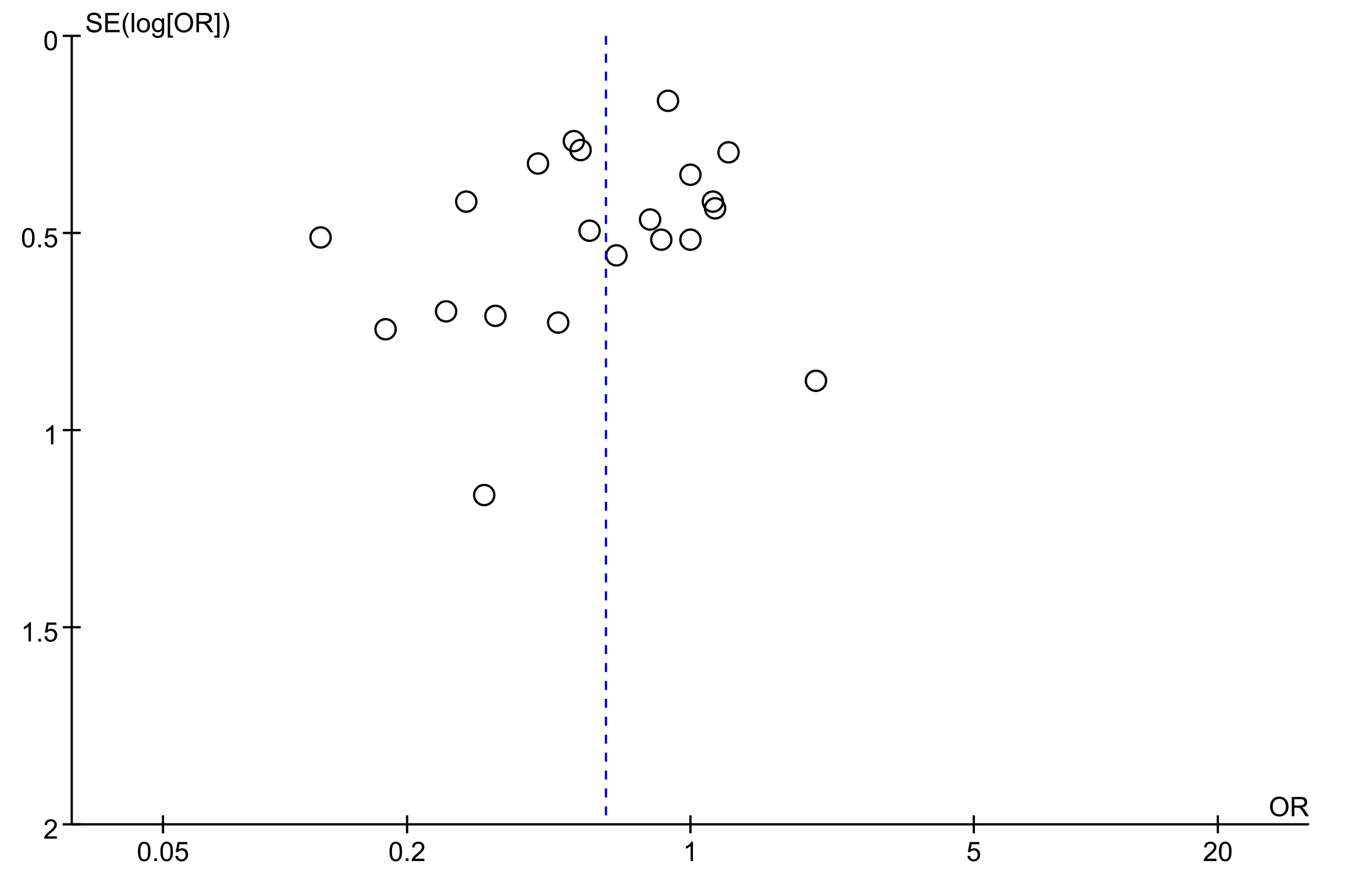

Supplement: Supplemental Material [file IRNF_A_2072338_SM1559.jpg]
