# Supplementary material for: Effect of goal-directed fluid therapy on renal function in critically ill patients: a systematic review and meta-analysis
Source: Ren Fail. 2022 May 10;44(1):777–89. doi: 10.1080/0886022X.2022.2072338 (PMC9103701; doi:10.1080/0886022X.2022.2072338)
Supplement: Supplemental Material [file IRNF_A_2072338_SM1547.docx]

**Supplementary Table 1. The outcome of acute kidney injury in the included studies**

| **Studies** | **Population** | **AKI in GDFT group n(%)** | **AKI in control group n(%)** | **AKI** **definition** | **Follow-up time** |
| --- | --- | --- | --- | --- | --- |
| McKendry et al.2004 | After cardiac surgery | 1(1.1) | 3(3.5) | Not mentioned | during the hospital stay |
| Lin et al 2006 | Septic shock | 42(38.9) | 64(55.2) | Not mentioned | during the ICU stay |
| Jhanji et al. 2010 | After major gastrointestinal surgery | 3(7) | 10(22) | The AKIN criteria | 7 days after surgery |
| Goepfert et al.2013 | After cardiac surgery | 3(6.5) | 8(1.7) | The AKIN criteria | during the hospital stay |
| Kanji et al. 2014 | Undifferentiated shock | 65(68) | 88(95) | The KDIGO criteria | 28 days |
| Pestaña et al. 2014 | After major abdominal surgery | 8(11.1) | 9(12.9) | At least a doubling of serum creatinine or oliguria (<500 mL/24 hours) | during the hospital stay |
| Pearse et al. 2014 | After major abdominal surgery | 17(4.6) | 17(4.7) | KDIGO criteria | 30 days after surgery |
| Suzuki et al. 2014 | After cardiac surgery | 9(17.0) | 12(26.7) | The RIFLE criteria | during the ICU stay |
| Thomson et al. 2014 | After cardiac surgery | 8(6.5) | 28(19.9) | The AKIN criteria | 3 days after surgery |
| Yealy et al. 2014 | Septic shock | 12(3.1) | 11(2.8) | The duration of dialysis during the acute hospitalization, truncated at 60 days, in patients who had not had a long-term need for dialysis before enrollment | 7 days |
| Liu et al. 2016 | Moderate brain injury& traumatic shock | 0(0) | 0(0) | Not mentioned | 48 hours |
| Jin et al. 2016 | After cardiac major vascular surgery | 32(24.4) | 38(37.6) | Not mentioned | during the ICU stay |
| Schmid et al. 2016 | After major abdominal surgery | 53(57.6) | 46(52.3) | KDIGO criteria | during the ICU stay |
| Luo et al. 2017 | After brain surgery | 3(4.1) | 6(8.3) | Not mentioned | 30 days |
| Huang et al. 2018 | Hypovolemic shock | 3(12) | 10(43.5) | Not mentioned | 72 hours |
| MacDonad et al.2019 | After major gastrointestinal surgery | 9(6.2) | 11(7.7) | KDIGO criteria | 30 days after surgery |
| Pan et al. 2019 | After cardiac surgery | 50(48.5) | 47(69.1) | KDIGO criteria | 72 hours after surgery |
| Musikatavorn et al. 2021 | Septic shock | 4(5) | 2(2.3) | KDIGO criteria | 72 hours |
| Parke et al. 2021 | After cardiac surgery | 96(26.8) | 105(29.4) | KDIGO criteria | during the hospital stay |
| Waal et al. 2021 | After high-risk abdominal surgery | 12(4.8) | 10(4.3) | Not mentioned | 30 days after surgery |
| Wang et al. 2021 | open abdomen patients | 6 (9.09) | 9 (13.24) | Not mentioned | 30 days after open abdomen |
| Froghi et al. 2022 | After liver transplantation | 16 (53.33) | 16 (53.33) | KDIGO criteria | hospital discharge |

## AKI, acute kidney injury; AKIN, Acute Kidney Injury Network; GDFT, goal-directed fluid therapy; ICU, intensive care unit; KDIGO, Kidney Disease: Improving Global Outcomes; n, number; RIFLE, Risk, Injury, Failure, Loss of kidney function and End-stage renal failure.
