# Supplementary material for: Effect of goal-directed fluid therapy on renal function in critically ill patients: a systematic review and meta-analysis
Source: Ren Fail. 2022 May 10;44(1):777–89. doi: 10.1080/0886022X.2022.2072338 (PMC9103701; doi:10.1080/0886022X.2022.2072338)
Supplement: Supplemental Material [file IRNF_A_2072338_SM1544.docx]

**MEDLINE (Via PubMed) and Cochrane search strategy**

#1 MeSH descriptor: [Intensive Care Units] explode all trees

#2 MeSH descriptor: [Emergency Medical Services] explode all trees

#3 MeSH descriptor: [Emergency Service, Hospital] explode all trees

#4 MeSH descriptor: [Critical Illness] explode all trees

#5 MeSH descriptor: [Critical Care] explode all trees

#6 ((intensive care) OR (ICU) OR (critical illness) OR (critically ill): OR (Critical Care) OR (emergency service) OR (Emergency Medical Services) OR (emergency department)): ti,ab

#7 #1 OR #2 OR #3 OR #4 OR #5 OR #6

#8 MeSH descriptor: [hemodynamics] explode all trees

#9 MeSH descriptor: [Fluid Therapy] explode all trees

#10 (haemodynamic*) OR (fluid therap*) OR (fluid administrat*) OR (fluid manag*) OR (fluid challeng*) OR (resuscitation) OR (intravenous fluid*): tw

#11 #8 OR #9 OR #10

#12 ((goal-directed) OR (goal-oriented) OR (target-directed) OR (GDT) or (GDFT) or (EGDT) or (protocol-based) or (protocol directed) or (?-guided)): ti,ab

#13 #11 and #12

#14 clinical trial.pt.

#15 exp Clinical Trials/

#16 "usual care": ti,ab

#17 "control*": ti,ab

#18 #13 OR #14 OR #15 OR #16

#19 #7 AND #13 AND #18

**Embase search strategy**

#9 #3 AND #6 AND #7 AND #8

#8 'clinical trial':pt OR 'clinical trial'/exp OR 'clinical trial':ti,ab OR 'control':ti,ab

#7 'goal-directed':ab,ti OR 'goal-oriented':ab,ti OR 'target-directed':ab,ti OR 'gdt':ab,ti OR 'gdft':ab,ti OR 'egdt':ab,ti OR 'protocol-based':ab,ti OR 'protocol

#6 #4 OR #5

#5 'hemodynamic*':ti,ab,kw OR 'fluid therap*':ti,ab,kw OR 'fluid administrat*':ti,ab,kw OR 'fluid manag*':ti,ab,kw OR 'fluid challeng*':ti,ab,kw OR 'resuscitation':ti,ab,kw OR 'intravenous fluid*':ti,ab,kw

#4 'hemodynamics'/exp OR 'fluid therapy'/exp

#3 #1 OR #2

#2 'intensive care':ab,ti OR 'icu':ab,ti OR 'critical illness':ab,ti OR 'critically ill':ab,ti OR 'critical care':ab,ti OR 'emergency service':ab,ti OR 'emergency medical services':ab,ti OR 'emergency department':ab,ti

#1 'intensive care units'/exp OR 'intensive care units' OR 'emergency medical services'/exp OR 'emergency medical services' OR 'emergency service, hospital'/exp OR 'emergency service, hospital' OR 'critical illness'/exp OR 'critical illness' OR 'critical care'/exp

**CBM search strategy**

#1 "重症医学科"[常用字段:智能] OR "急诊"[常用字段:智能] OR "ICU"[常用字段:智能] "重症监护"[常用字段:智能]

#2 "液体治疗"[常用字段:智能] OR "容量管理"[常用字段:智能] OR "液体复苏"[常用字段:智能]

#3 "目标导向"[常用字段:智能] OR "目标指导"[常用字段:智能]

#4 #1 AND #2 AND #3
